# Lignosulfonate-Based Polyurethane Adhesives

**DOI:** 10.3390/ma14227072

**Published:** 2021-11-21

**Authors:** Sandra Magina, Nuno Gama, Luísa Carvalho, Ana Barros-Timmons, Dmitry Victorovitch Evtuguin

**Affiliations:** 1 CICECO—Aveiro Institute of Materials and Department of Chemistry, University of Aveiro, 3810-193 Aveiro, Portugal; smagina@ua.pt (S.M.); nuno.gama@ua.pt (N.G.); anabarros@ua.pt (A.B.-T.); 2 LEPABE—Laboratory for Process, Environmental and Energy Engineering, Faculty of Engineering, University of Porto, 4200-465 Porto, Portugal; lhcarvalho@estv.ipv.pt; 3 DEMad—Department of Wood Engineering, Polytechnic Institute of Viseu, 3504-510 Viseu, Portugal

**Keywords:** lignosulphonate, adhesive, pMDI, polyurethane, adhesion, curing kinetics, PEG, DSC, ABES

## Abstract

The feasibility of using lignosulfonate (LS) from acid sulphite pulping of eucalyptus wood as an unmodified polyol in the formulation of polyurethane (PU) adhesives was evaluated. Purified LS was dissolved in water to simulate its concentration in sulphite spent liquor and then reacted with 4,4′-diphenylmethane diisocyanate (pMDI) in the presence or absence of poly(ethylene glycol) with *M*_w_ 200 (PEG_200_) as soft crosslinking segment. The ensuing LS-based PU adhesives were characterized by infrared spectroscopy and thermal analysis techniques. The adhesion strength of new adhesives was assessed using Automated Bonding Evaluation System (ABES) employing wood strips as a testing material. The results showed that the addition of PEG_200_ contributed positively both to the homogenization of the reaction mixture and better crosslinking of the polymeric network, as well as to the interface interactions and adhesive strength. The latter was comparable to the adhesive strength recorded for a commercial white glue with shear stress values of almost 3 MPa. The optimized LS-based PU adhesive formulation was examined for the curing kinetics following the Kissinger and the Ozawa methods by non-isothermal differential scanning calorimetry, which revealed the curing activation energy of about 70 kJ·mol^−1^.

## 1. Introduction

Wood-based composites cover a variety of products, from fiberboards to laminated beams, and are used for numerous non-structural and structural applications such as panels for interiors, furniture, support structures in buildings, etc. Wood raw-materials used in the production of wood-based composites include fibers, particles, flakes, veneers, laminates, or lumber [1,2]. In most conventional wood-based composites, adhesive bonding is achieved by non-renewable petroleum-derived thermosetting synthetic resins including phenol-formaldehyde (PF), urea-formaldehyde (UF), melamine-formaldehyde (MF), polyurethanes (PUs) and polymeric diphenylmethane diisocyanate (pMDI). Several auxiliary chemicals are also added to plasticize adhesive polymers, enhance tackiness, improve heat resistance, or lower costs [1,3,4]. Due to the waning of petrochemical supplies but also due to the public awareness related to the environment and its protection, as well as governmental regulations, the use of bio-based adhesives in wood and fiber composites has attracted scientific and economic interest for the last several decades [4,5,6,7]. 

Within the diverse synthetic resins aforementioned, PUs are versatile designer polymers that display varied properties being adjustable to a wide range of applications namely foams (rigid and flexible), elastomers, paints and coatings, adhesives, and even for medical applications [8,9,10,11]. The global PU market size was valued at USD 70.67 billion in 2020 and is expected to grow at a compound annual growth rate (CAGR) of 3.8% from 2021 to 2028 [12]. Typically, PUs are prepared through the addition of isocyanates (comprising more than one reactive isocyanate group per molecule) and polyols (containing two or more reactive OH groups per molecule) yielding polyurethane linkages in the polymer backbone [8,9,10,11]. While isocyanates are very harmful and, at the end of their life cycle, PUs may release toxic compounds such as amines leading to health and environmental concerns, so far it has been very difficult to replace them. Furthermore, the implementation of regulations by some governments have motivated both academia and industry to develop safer and “greener” alternative routes to produce more environmentally friendly PUs. To reduce the environmental impact of PUs, efforts have focused on the replacement of fossil derived polyols by bio-based ones, such as castor oil [13,14,15,16,17,18,19] and other vegetable oils [20,21,22], crude glycerol [15,23], lignin [15,24,25], among others.

Technical lignins can act as macropolyols in PU synthesis due to high amount of phenolic and aliphatic hydroxyl moieties in their structure [24,25,26,27,28,29,30]. The direct exploitation of technical lignins, as polyols or blending with industrial polyols, is energetically and environmentally advantageous [31] and the ensuing biomass-based PUs are more biodegradable than those derived from petroleum-based polyols [32]. Hence, lignins can be used as such, or after chemical modification to obtain a more reactive lignin, alone or in combination with other polyols [24,27,28,29,30,33,34,35]. It is well known that lignins (in general) act both as a network former (due to its functionality being higher than 2) and as a reinforcing component in PU formulations. The latter is mainly due to the high content of condensed aromatic rings in lignin contributing to its generally stiff structure. Therefore, to counterbalance the stiff character of the lignin macromolecules, other polyols can be used as soft segments, such as poly(ethylene glycol) (PEG) and poly(propylene glycol) (PPG) [16,29,36,37], or bio-based polyols, such as castor oil [15,16,17,18,19], crude glycerol [15] and poly(ε-caprolactone) (PCL) [38] yielding grafted or cross-linked PUs with the possibility of controlling their flexibility and/or rigidity. Due to the high content of reactive aliphatic OH in lignins, for instance in organosolv lignins, these can be used as macromonomers without further chemical modification [34,35]. However, considering that the structure of technical lignins is extremely dependent on the plant source from which it is obtained, the final properties of the lignin-based PUs depend highly on the lignin’s plant source [39]. The low reactivity of the lignin macromonomer toward isocyanates is usually related to the fact that only a certain proportion of the total OH groups can react (due to steric hindrance from the highly branched three-dimensional structure of lignin and intramolecular hydrogen bonding) yielding products without desirable performance [39,40,41]. From several studies in the literature around 20 wt.% of unmodified lignin is the limit for lignin incorporation in PU formulations [28]. 

Another important issue for the adhesive industry is the control of the curing process since it affects the final properties of the material. This requires knowledge of the kinetics of the curing process. In a previous work, Gama and co-workers [13] studied the curing process of castor oil-based PU adhesives by differential scanning calorimetry (DSC) using the two most known approaches, the Kissinger [42] and the Ozawa [43] methods. Both methods are rapid, easy to use and depend on a series of experiments based on heating samples at several different heating rate [44]. Kinetic parameters, such as the activation energy (*E*_a_) of the cure and the degree of cure (or conversion, α), were determined from non-isothermal measurements using different heating rates (*β*). α varies from 0 to 1, i.e., from completely uncured to fully cured, and can be determined by Equation (1).

(1) $$\alpha =H_{\left(t\right)}/H_{T}$$

where *H_(t)_* is the enthalpy of the reaction up to time *t* and *H_T_* is the total enthalpy of the reaction. Additionally, the rate of cure (*dα*·*dt*^−1^) is proportional to the rate of the heat generated and can be determined by Equation (2).

(2) $$d\alpha /dt=1/H_{T}\times dH_{\left(t\right)}/dt$$

when the curing rate (*β*) is increased, the peak temperature (*T_p_*) shifts to a higher temperature range, and this can be used to calculate *E_a_*, using methods such as the Kissinger method or Ozawa method [44], according to Equations (3) and (4), respectively.

(3) $$\ln(\beta /T_{p}^{2})=C-E_{a}/\left(RT_{p}\right)$$

(4) $$\ln(\beta )=C-E_{a}/\left(RT_{p}\right)$$

where *C* is a constant derived from *E*_a_, the kinetic constant (*k*_0_) and the universal gas constant (*R*). By plotting ln(*β*/*T*_p_^2^) vs. *1*/*T*_p_ and ln(*β*) vs. *1*/*T*_p_, the value of *E*_a_ can be determined. 

The ultimate goal of this work is to use industrial sulphite spent liquor (SSL) as a bio-based polyol to substitute petroleum-based polyols in the formulation of bio-based PU adhesives and also to reduce costs associated with LS purification. SSL from acid sulphite pulping of hardwoods, such as eucalypt, is mainly composed of LS, xylo-oligosaccharides and contains high amounts of pentose sugars, extractives of polyphenolic origin and inorganic salts [45,46,47]. To avoid the eventual participation of other components from SSL in the PU synthesis, LS was purified by dialysis and contained minimal organic/inorganic impurities. Therefore, in the adhesive preparation unmodified purified eucalyptus LS was used as bio-based polyol, water as LS solvent, PEG_200_ as a soft segment co-polyol and pMDI as a basic crosslinker. Since lignins generally contribute to PU stiffness, the addition of PEG with *M*_w_ 200 (PEG_200_) allows tuning the viscoelastic properties of PUs. Therefore, the variation in PEG_200_ content was studied and the final adhesion properties of the resulting adhesives were evaluated. Structural and thermal characterization was performed on the most relevant adhesive formulations. Finally, the kinetic parameters of the curing process were assessed for the most promising adhesive formulations. 

## 2. Materials and Methods

### 2.1. Materials and Reagents

**Figure 1:**
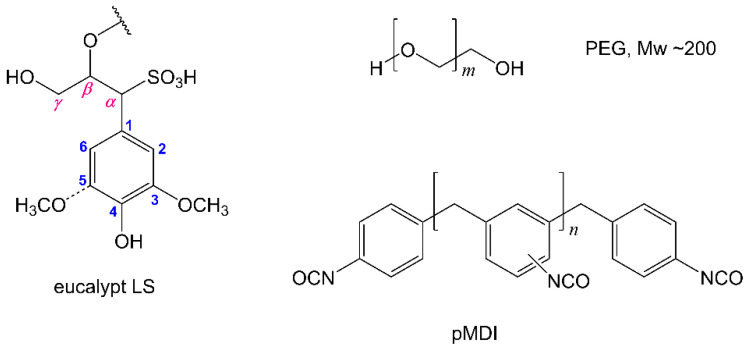
Conventional structural representation of eucalypt LS showing phenolic and aliphatic OH groups, PEG_200_ and commercial polymeric isocyanate pMDI.

Lignosulfonates (LS) were purified by dialysis in water for 24 h from thick spent liquor of the industrial magnesium-based acidic sulphite pulping of *Eucalyptus globulus* wood, which was supplied by Caima Company (Caima-Indústria de Celulose S. A., Constância, Portugal). The detailed purification and characterization of purified LS procedures were previously reported elsewhere and showed that it contained 17.1 wt.% of HSO_3_ groups, 2.4 wt.% of phenolic and 5.5 wt.% aliphatic OH groups [48]. Dibutyltin dilaurate (DBTDL) was purchased from Sigma-Aldrich Chem. Comp. (Madrid, Spain). PEG with average *M*_w_ 200 (PEG_200_) was supplied by Acros Organics (Lisbon, Portugal). Oligomeric isocyanate 4,4′-methylene diphenyl diisocyanate (pMDI) (Voranate M229) with 31.1% of NCO, a functionality of 2.7, a viscosity of 190 mPa·s (at 25 °C) and an isocyanate equivalent of 135 (values provided by the supplier) was kindly supplied by Dow Chemicals (Estarreja, Portugal). Commercial poly(vinyl acetate) (PVA) glue (white glue) was purchased in the local market. The structures of the main components of the synthesized PU formulations are depicted in Figure 1.

### 2.2. LS-Based Adhesive Synthesis

For all formulations, purified LS powder (500 mg) was first dissolved in water (400 μL) or a mixture of water with PEG_200_ (0, 50, 100 and 150 μL) to form the base solution, in a glass vessel with screw cap with a magnetic stirring bar, at room temperature. The volume of water used was initially optimized to ensure full solubilization of LS and ease of stirring. The mixture was kept under constant magnetic stirring for 5 min and then 50 μL of DBTDL was added as the catalyst. Finally, 900 or 1000 mg of crosslinker pMDI was introduced in the reaction mixture and the reaction proceeded for a certain time period ranging from 30 s to 5 min depending on the application. For all formulations, the quantities of LS, water and DBTDL were kept constant (500 mg, 400 μL and 50 μL, respectively) while only the contents of PEG_200_ and pMDI were varied, according to the formulations presented in Table 1. The molar ratio NCO/OH was calculated based on the contribution of the total amount of OH groups in LS [48] and OH groups in PEG_200_.

For the study of the curing process using DSC and DMA, the stirring time was 30 s. For the preparation of the samples used for FTIR and TGA analyses, a 5 min stirring was performed after which the adhesive was left curing for 48 h at room temperature. The same procedure was followed for samples submitted to the ABES testing. 

### 2.3. Adhesive Characterization

LS-based PU adhesives were characterized by Fourier transform infrared spectroscopy (FTIR) using a FTIR System Spectrum BX (PerkinElmer, Waltham, MA, USA), coupled with a universal ATR sampling accessory, in absorbance from 4000 to 500 cm^−1^ with a 4 cm^−1^ resolution. Adhesive samples were analyzed as such after complete curing (48 h at room temperature), 128 scans were averaged, and all spectra were baseline corrected and normalized (by the min-max normalization technique [49]) for further analysis. 

Thermogravimetric analysis (TGA) of the samples was carried out using a Setsys Evolution 1750 TGA-DSC thermogravimetric analyzer (Setaram, Caluire, France) equipped with a DSC plate rod accessory. Samples were analyzed from room temperature to 800 °C at a heating rate of 10 °C·min^−1^ under nitrogen flow rate of 200 mL·min^−1^ using an alumina crucible. 

Dynamic mechanical analysis (DMA) of the polymer films was carried out using a Tritec 2000 DMA instrument (Triton Technology, Leicestershire, UK). The LS-based PU formulation before curing was studied as such using a stainless-steel material pocket accessory [50] in single cantilever bending mode. A first temperature scan for the LS-based formulation curing process was carried out from room temperature up to 180 °C at a heating rate of 5 °C·min^−1^, with a displacement of 0.020 mm and at a frequency of deformation (oscillating frequency) of 1 Hz. Next, the same material pocket containing the cured LS-based PU was used to perform a second temperature scan in order to determine its *T*_g_. This second DMA run was carried out from −20 °C up to 200 °C (before degradation) at a heating rate of 2 °C·min^−1^, with a displacement of 0.020 mm and at a frequency of 1 Hz.

Differential scanning calorimetry (DSC) analysis was carried out on a Power Compensation Diamond DSC (PerkinElmer, Waltham, MA, USA) previously calibrated using the melting points of indium and lead as standards for temperature calibration and the heat of fusion of indium as standard for heat calibration [51,52]. The LS-based PU formulation samples (around 5–10 mg) were encapsulated in hermetically sealed stainless-steel pans that can withstand a maximum internal pressure of 24 bar. Dynamic (non-isothermal) DSC runs were performed in the temperature range from −10 to 150 °C, at different heating rates, namely 5, 10, 15 and 20 °C·min^−1^. 

**Figure 2:**
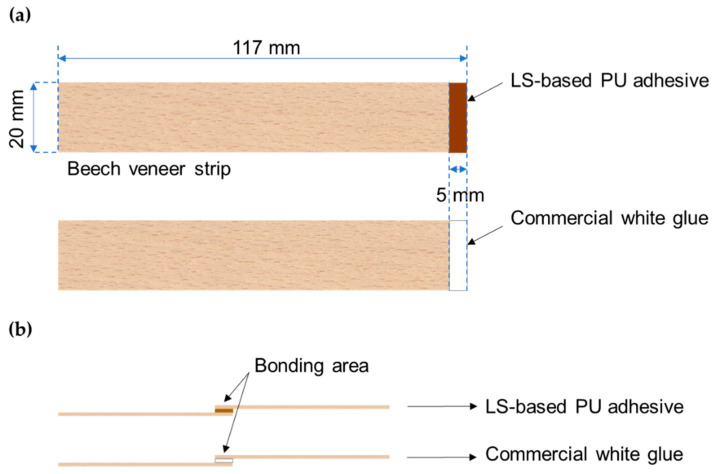
Schematic representation of assembly for the adhesive test: (**a**) the beech veneer strips with applied adhesive and (**b**) the final set of two bonded beech veneer strips.

The strength development of LS-based adhesives was assessed using the automated bonding evaluation system (ABES, Corvallis, OR, USA) at the Department of Wood Engineering, Polytechnic Institute of Viseu. Tests with ABES apparatus were carried out using beech (*Fagus sylvatica*) veneer strips (with a dimension of 117 mm × 20 mm and thickness of 0.5 mm). For each test, a new LS-based adhesive sample formulation was prepared. After adding pMDI (according to the synthesis method), the formulation was mixed using a magnetic stir for 5 min. Afterwards, 10 mg of pre-cured adhesive was applied and evenly distributed on the standard configuration of the beech veneer (over 5 mm of the edge of the beech veneer strips to cover the bonding area of 100 mm^2^), according to Figure 2. Then, a wood strip without adhesive was overlapped over the one with adhesive making sure the two strips were aligned giving an overlapping area of 100 mm^2^ (20 mm × 5 mm). This strip was glued to another strip in the same configuration and a 500 g load (49 kPa) was placed on top of the jointed wood strips. The structure was kept in this form for 24 h at room temperature for adhesive curing and the strips to be glued. For each formulation, a minimum of at least three sets of bonded strips were prepared.

## 3. Results and Discussion

**Figure 3:**
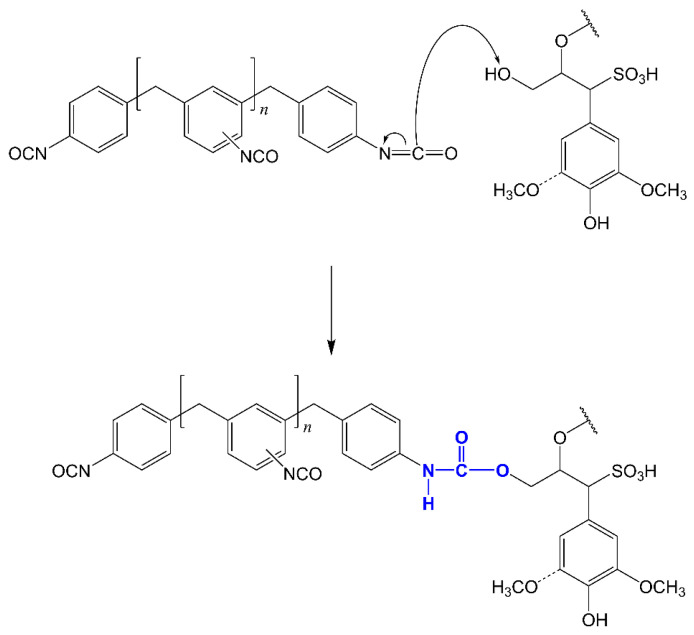
Schematic representation of the reaction between NCO group in pMDI and aliphatic OH in the γ–position of eucalypt LS.

The polycondensation of unmodified lignins with isocyanates with large molecular structures such as pMDI is unlikely to occur without the addition of catalysts due to the lack of reactivity, which is related to the steric hinderance of OH groups in lignin and of NCO groups in pMDI as well as low diffusion due to the high viscosity of the reaction medium [34]. Accordingly, DBDTL was used as a catalyst. It is assumed that in LS, as in other technical lignins, the primary OH groups are the most reactive with isocyanate moieties [34]. This assumption is reflected in the reaction scheme proposed in Figure 3, where the most reactive OH groups in the γ position of the lignin structural unit react with pMDI. It is noteworthy that steric hindrance of phenolic OH groups is much more pronounced in hardwood than in softwood lignin, because the former has quite a high ratio of syringyl (S) over guaiacyl (G) structural units [53,54]. In fact, the eucalyptus LS used in this work had the particularly high S:G ratio of 78:22 [48]. Furthermore, in the presence of water, the reactivity of pMDI with lignin is hampered by isocyanate competition reactions with the formation of the corresponding amines [6,10,14]. Therefore, it can be expected that a polymeric network of low crosslinking will result from the reaction of pMDI and LS alone. However, the addition of highly reactive water-soluble polyol to the reaction system can increase crosslinking between polymer chains and, if a polyol is polar enough to improve segmental movement of the resulting network, it can positively contribute to the adhesive properties of the final synthetic glue. Based on these considerations, a series of formulations were synthesized (Table 1) using, in addition to pMDI and LS, low molecular weight polyethylene glycol diol (M_w_ 200). The produced LS-based adhesives were structurally and thermally characterized and the adhesion strength have been evaluated.

### 3.1. Chemical and Thermal Characterization of LS-Based PU Adhesives

**Figure 4:**
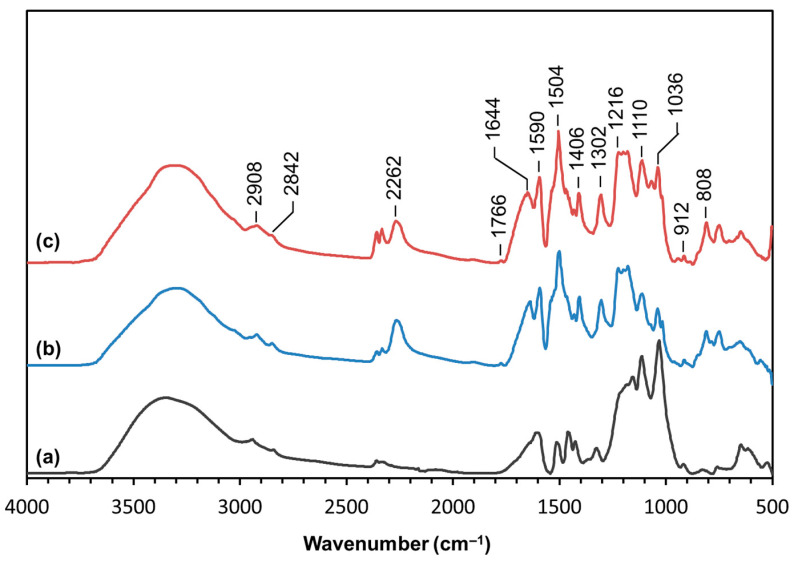
Normalized FTIR-ATR spectra of (**a**) LS, (**b**) LS-based PU adhesive (formulation 5 in Table 1), and (**c**) LS-based PU adhesive with PEG_200_ (formulation 8 in Table 1).

The parent LS and typical adhesive formulations involving LS and pMDI (LS-MDI) and LS, pMDI and PEG_200_ were structurally characterized by FTIR-ATR. Although semiquantitative, this technique allows for rough conclusions. The corresponding normalized spectra of LS, LS-based adhesive without (LS-MDI) and with PEG_200_ (LS-MDI-PEG) are presented in Figure 4 and the bands assignment are listed in Table 2. The reaction of OH groups in LS with pMDI in the PU samples produced was confirmed by the decrease in the intensity of the band peaked at 3430 cm^−1^ (δ O–H) and the band at 1036 cm^−1^ (δ C–OH) [45,55,56]. Simultaneously, the newly formed bands in the PU samples were observed at 1766 (very slight), 1504 (the most evident), 1406 (also significant) and 1216 cm^−1^, and assigned to the stretching vibration in urethane groups [57]. Noteworthy that the band at 1504 cm^−1^ in PU is the result of superposition of lignin aromatic band and the secondary NH band, which shoulder is clearly observed at ca. 1530 cm^−1^. Meanwhile, the characteristic bands at 2262 (C–N stretching), 1644 (C=O stretching) and 1590 cm^−1^ (N–H deformations) [57] are assigned to unreacted isocyanate groups from pMDI still present in the PU product suggesting that the PU curing process was not complete. This is in agreement with the formulation’s composition (Table 1) as NCO groups were used in excess amount. It is noteworthy that the integral intensity of the isocyanate band at 2262 cm^−1^ was less to ca. 30% for LS-MDI-PEG than for LS-MDI (Figure 4), thus confirming the importance of adding PEG_200_ to obtain more extensive crosslinking of the molecular network.

**Figure 5:**
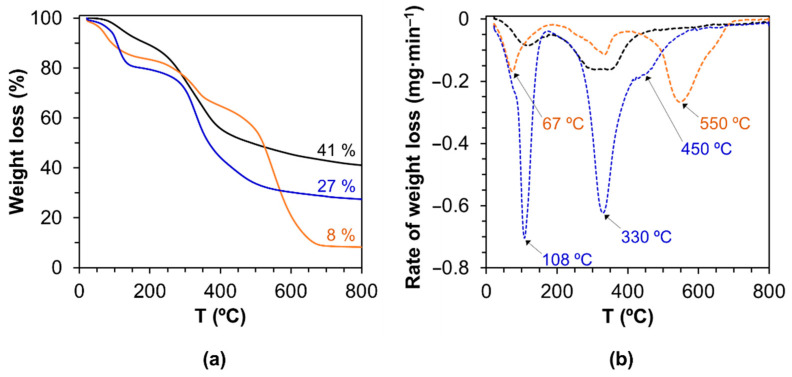
TGA curves of LS (**― ---**), LS-based PU without PEG_200_ (formulation 5; **― ---** LS-PU), and LS-based PU containing PEG_200_ (formulation 8; **― --- **LS-PEG-PU): (**a**) weight loss under inert N_2_ gas flow and (**b**) derivative of the weight loss.

The thermal behavior of LS, LS-PU (formulation 5 in Table 1) and LS-PU-PEG (formulation 8 in Table 1) are different from each other (Figure 5a). Under inert atmosphere (N_2_ flow), LS undergoes a first weight loss with a maximum loss at around 120 °C (Figure 5b), related to the release of moisture followed by the degradation of functional groups (around 330 °C, Figure 5b), such as sulphonic groups, and the release of low molecular mass products [59,60] giving rise to a quite high char content (41%). LS- PU and LS-PEG-PU also undergo a first weight loss around 60–100 °C (Figure 5b) related mostly to the release of moisture.

The degradation of PUs can usually be divided into two major stages. The first stage is dominated by the degradation of the hard segments, while the second stage is controlled by the soft segments (polyol) [13,61]. Yet, all thermograms present a weight loss at lower temperatures attributed to the loss of water. This was expected since the adhesives were prepared in aqueous media. Regarding LS-PU, the first stage with a maximum weight loss at 335 °C is most likely related to the decomposition of urethane moieties in the PU and other functional groups from LS, the second slow weight loss stage with a maximum weight loss at 550 °C was attributed to further structural rearrangements of pMDI and LS counterparts during char formation [25]. Regarding LS-PEG-PU, the weight loss registered below 100 °C even higher than for LS-PU in agreement with the fact that water interacts strongly with PEG so it was not efficiently removed during the cure. In what concerns the degradation of the polymeric material, the first stage with a maximum weight loss at 330 °C is related to the decomposition of urethane groups and LS (similarly to LS-PU) but, in this case, the second stage is more intense and a shoulder appears around 450 °C in Figure 5b. Curiously, no mass loss is detected at higher temperature like for LS-PU. These differences are most probably associated with the presence of PEG [61]. Under inert gas, PEG_200_ displays a single stage decomposition with a maximum weight loss at 290 °C (Figure S1, Supplementary Materials). Hence, the decomposition of PEG_200_ contributed to the increased intensity of the degradation peak at ca. 330 °C. Furthermore, considering the presence of the shoulder at 450 °C and the absence of degradation at higher temperature, as well as the higher amount of char formed, it is clear that the presence of PEG_200_ in LS-PEG-PU results in a very distinct degradation pathway. Significantly higher residual char content in the thermal degradation of LS-PEG-PU than LS-PU (Figure 5) is indicative of the much denser molecular structure of the former. This is in tune with FTIR analysis results that showed the eventual intensification of molecular crosslinking in LS-pMDI adhesive with addition of PEG_200_ to the reaction system. 

**Figure 6:**
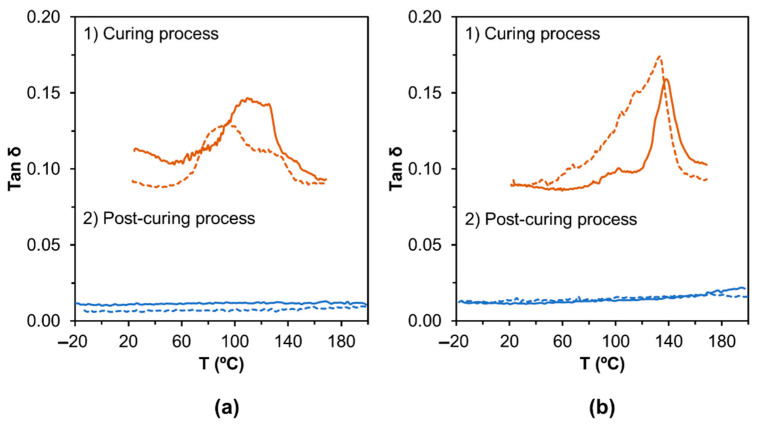
DMA profiles of (**a**) LS-based PU without PEG_200_ (LS-PU) and (**b**) LS-based PU with PEG_200_ (LS-PEG-PU). Full line and dashed lines are duplicates of the same formulation.

The curing of LS-based PU formulation with and without the addition of PEG_200_ was studied by DMA analysis under non-isothermal conditions, as depicted in Figure 6a(1),b(1). A post-curing heating scan was carried out to determine the *T*_g_ of both LS-based PU adhesives (Figure 6a(2),b(2)). For each PU formulation, a duplicate was prepared and analyzed. It should be noted that the curves of the storage and loss modulus, *E*′ and *E*″ respectively, are not shown due to the influence of the material pocket. 

Concerning the curing process, in the tan *δ* profile the transition assigned to the PU curing starts around 60 °C for both types of LS-based PU formulations with and without PEG_200_, i.e., formulations 5 and 8 (Table 1), respectively. To study curing processes using DMA analysis, two points should be determined, the point of gelation (when the material changes from a viscous liquid to a viscoelastic solid, which is the *E*′–*E*″ crossover or where tan *δ* is equal to one) and the point of vitrification (when the curing system reaches such high viscosity that limits further curing and the bulk reaction stops, commonly taken as the onset plateau of the storage modulus) [62]. However, in this case, this type of study could not be carried out since the *E*′ and *E*″ curves could not be considered due to the use of the material pocket accessory. Still, some observations can be pointed out when comparing the DMA profile of each formulation with its respective duplicate. Taking into account that each formulation was replicated using exactly the same procedure, it is clear that the curing behavior of two equal PU formulations does not match (the profile has some similarities but is still different). The only possible explanation for this occurrence is the mixing process after the addition of crosslinker pMDI and consequently the reagents diffusion. In the formulation without PEG_200_, isocyanate groups react with water and OH groups in LS (most likely aliphatic OH groups in the *γ*–position [34]), while in the formulation with PEG_200_, isocyanate groups react with water, OH groups in LS and OH groups from PEG_200_. Reactions between the isocyanate groups with available OH groups depend mostly on the accessibility/reactivity of different OH groups and also on the efficiency of the mixing process and consequently diffusion. Therefore, due to the high apparent viscosity of the reaction mixture, it is possible that the mixing was not perfect enough and that the extent of crosslinking was different for the same formulation affecting the curing process. In fact, the addition of PEG_200_ to the formulation also changed the curing profile (Figure 6b(1)) compared to the one observed for the LS-based PU without PEG_200_. In this case, the temperature at which the curing process appears to be completed increased to around 135–140 °C probably due to a chain entanglement effect and subsequent diffusion limitation of reactive moieties [63,64]. Moreover, the broadening of tan *δ* peak for the system containing PEG_200_ seems to be less pronounced indicating a more uniform crosslinked network.

Since DMA is much more sensitive to detect *T*_g_ than other techniques, such as DSC, and can easily measure transitions that may not be apparent in other thermal methods [62], after the curing process, each material pocket containing the cured PU adhesive was cooled back to −20 °C and was used to perform a second DMA run to determine the *T*_g_. Based on TGA analysis (Figure 5), this run was carried out only up to 200 °C to avoid potential thermal degradation of the products. Surprisingly, no *T*_g_ was observed in any post-curing tan δ profile, which means that LS-based PU adhesives do not display clear softening behavior in the temperature range between −20 and 200 °C. The values of *T*_g_ are dependent on the lignin and PEG contents and usually increase with increasing weight fraction of lignin (stiff component) and decrease with increasing weight fraction of PEG (soft segment) [34,65]. Though most values reported in the literature (ranging from −40 to 105 °C [34,65]) were determined by DSC and values determined from DMA analysis can be higher than DSC’s by 25 °C [62], some softening behavior would be expected. 

### 3.2. Evaluation of the Adhesion Strength of LS-Based PU Adhesives

**Figure 7:**
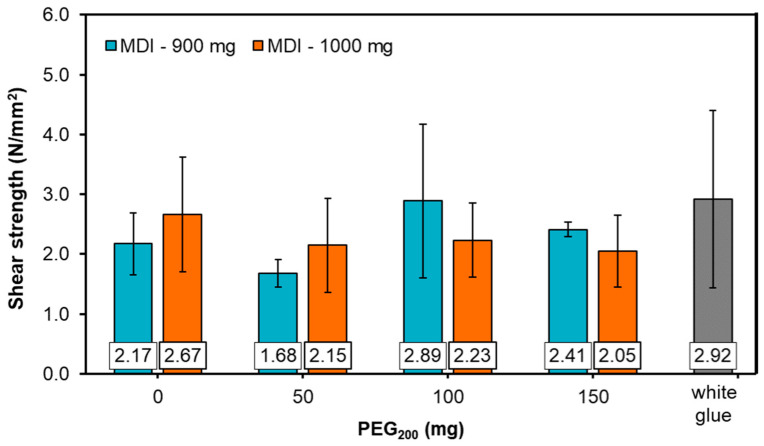
Shear strength values for each LS-based PU formulation as a function of the content of PEG_200_ against the shear strength of commercial white glue (each value corresponds to the average of a minimum of three values).

It is known that an efficient wood adhesive must spread across the wood surface but also wet the said surface to increase the contact area, i.e., the interface. Hence, the goal is to develop molecular interactions between the adhesive and wood. Therefore, a good adhesive wetting can produce effective wood bonding. Yet, other parameters are also important such as efficient solidification of the adhesive to provide strength (in this case, through chemical curing) and sufficient deformability (related to the flexibility of the resulting adhesive due to the addition of PEG_200_) of the cured adhesive to reduce stress [66]. ABES testing was performed to evaluate the strength of adhesion of the LS-based PU adhesives and to assess the effect of the PEG_200_ amount on the adhesion results. Additionally, results were compared with those obtained using a commercial white glue (Figure 7). ABES testing measures the force in tensile mode needed to break the adhesive bond and shear strength gives an indication of the strength of an adhesive. Therefore, the higher is the force needed to break the bond, the higher is the shear strength value. At first glance, all LS-based PU adhesives showed adhesion strengths somewhat lower or similar to those obtained using commercial white glue. This means that LS-based PUs displayed variable adhesive properties depending on the formulation composition, but still comparable to the commercial adhesive. Another fact is that the errors associated with each strength value are quite high. This was especially noticeable for the commercial white glue. Considering that one duplicate of each formulation was prepared, the results suggest that there are significant differences between duplicates of the same formulation, as observed when performing DMA analysis, affecting crosslinking and thus the curing process and eventually the adhesion strength. Furthermore, for each formulation, a minimum of three sets of bonded strips were prepared and even within the same formulation, different strength values were obtained (yet, only concordant values were chosen and individual data are not shown). This indicates that for the same formulation, the samples applied on the wood strip are different from one another, possibly due to poor reagents mixing. Furthermore, only 10 mg of adhesive was applied to the strip, which may not be fully representative of the entire product. It could be also suggested that comparing formulations containing 900 mg of pMDI with those containing 1000 mg of pMDI, strength values are quite similar. However, it appears that the strength values of formulations containing 1000 mg showed less variability than those of formulations containing 900 mg of pMDI. These results suggest that when the system comprise higher amounts of pMDI it is under kinetic control (higher reactivity, hence more reticulation) as opposed to diffusion control (which leads to higher heterogeneity, hence less reticulation) when lower amounts of pMDI are used. As expected, the best adhesion results were obtained with the addition of 100 μL of PEG_200_ as this polyol provides flexibility and promotes further crosslinking extension as discussed above.

Indeed, in practice, when adhesives were prepared, formulations containing higher amount of pMDI and also containing PEG_200_ were easier to homogenize. Overall, adhesion strength results showed some effect of the variation in pMDI content and the amount of added PEG_200_. In particular, with a lower content of pMDI in the reaction mixture, the shear stress showed a maximum with the addition of PEG_200_ (100–150 mg, Figure 7). This is not particularly surprising since, as suggested by Thring and co-workers [67], the chain length of PEG_200_ may be too short to bridge in the network due to steric hindrance from the chemical structure of lignin. Therefore, a balance must be found between the lignin content and PEG_200_ since lignin-derived PU materials produced using PEG_200_ are either too weak at low lignin content, or too brittle at higher amounts of lignin. In this case, it is possible that PEG with higher molecular weight could have provided a different adhesion behavior.

**Figure 8:**
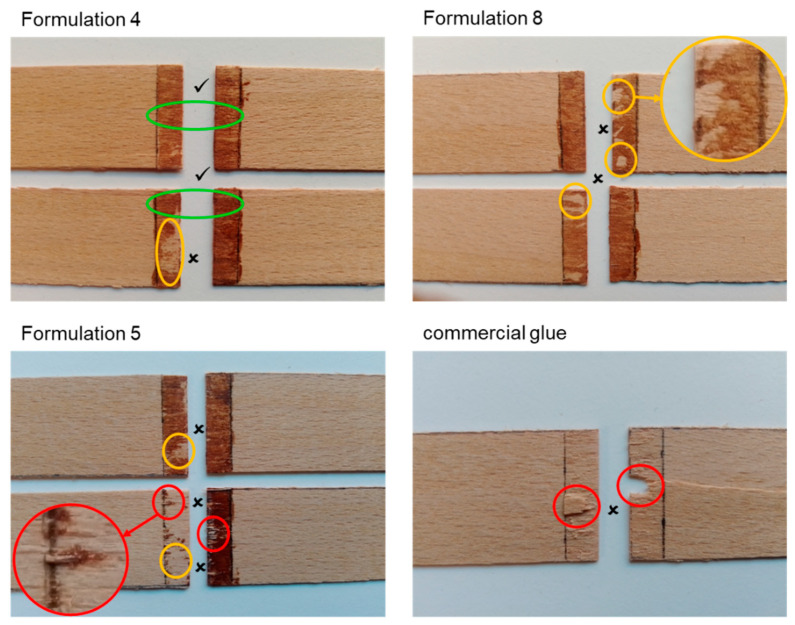
Photographs of selected beech veneer strips after ABES testing showing different types of failure (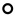 Adhesion failure within the adhesive—good result 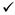; 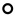 Adhesion failure to the substrate—failed result 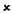; and 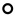 substrate failure—failed result 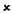).

It is noteworthy that when performing ABES testing, the way adhesive bonding breaks is extremely important. Figure 8 depicts some photographs of the bonding areas of some wood strips after ABES testing. Since the main objective of ABES testing is to determine the adhesive strength, the adhesive failure must occur within the adhesive (as is shown by green circles when adhesive remained in both strips), but not adhesive failure with adhesion to the substrate (as is shown by yellow circles when the adhesive was transferred to one of the strips due to adhesion failure between adhesive and substrate). Additionally, substrate failure (red circles) should not occur neither though, when it does, it means that the strength of the adhesive is too high [68]. Based on the results obtained, the occurrence of adhesion failure to the substrate suggests that the LS-based PU adhesive did not sufficiently wet the strip surface. Adhesives must flow to the surface of the wood and penetrate the entire tissue of the wood so that intermolecular interactions (strong covalent bonds and/or mechanical locking) between the adhesive and the wood can occur [66]. In fact, all adhesives lacked sufficient fluidity and low viscosity to be sufficiently absorbed by the wood strip. In addition, the bonding failure of the adhesives was very irregular confirming the lack of homogeneity in the composition of each LS-based PU formulation. In practice, optimization of the adhesive composition in relation to the bonded substrate is still required.

According to our observations, the addition of PEG_200_ favored better adhesion of the adhesive to the substrate (wood strips). These adhesive formulations (e.g., formulations 3, 4, 7, and 7) revealed also the highest values of shear stress (Figure 7). Apparently, the improved crosslinking during curing of the PU adhesive with the proper proportion of LS, pMDI and PEG_200_ (e.g., formulation 3, Table 1) contributed positively to the adhesive strength in bulk.

### 3.3. Kinetic Study of the Curing Process of LS-based PU Adhesive Containing PEG_200_

**Figure 9:**
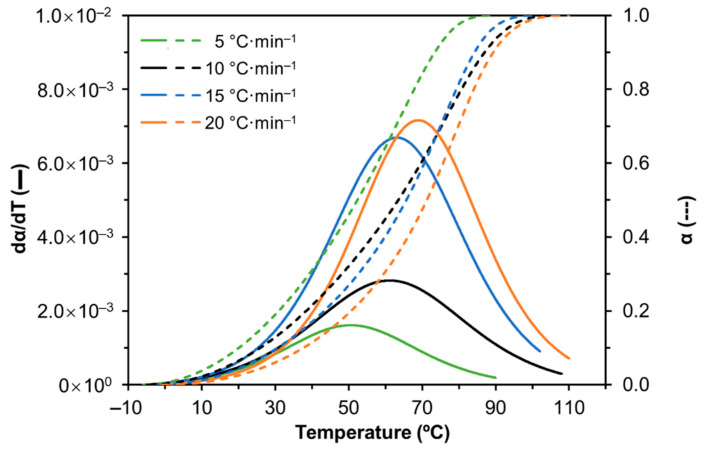
Effect of heating rate on the cure of LS-based PU containing 150 μL PEG_200_ (formulation 8, Table 1).

The study of the curing process is highly important in the industry as it is a complementary analysis tool that provides additional understanding on the mechanism of the curing reaction, in order to control and optimize the curing process. Therefore, the cure of LS-PEG_200_(150)-PU (formulation 8), which is the most promising and flexible, was studied using non-isothermal DSC. The plots of the degree of cure (conversion, α) and the rate of heat generated (dα/dT) at different heating rates (β) as a function of the temperature (*T*) are depicted in Figure 9. As expected [13,69], the conversion rate increased up to a maximum value and then decreased indicating that the free volume between the macromolecules allows molecular movement during the curing process up to 70 to 80% of conversion but beyond crosslinks start to break down. Furthermore, peaks shifted toward higher temperatures as β increased. As the temperature increased, α also increased up to 1 (maximum degree of conversion) slowly at the initial curing stage and then more abruptly at the end. The S-shaped curves confirmed that the reaction only starts after a certain temperature is achieved and this occurrence is in agreement with the literature [13,70].

**Figure 10:**
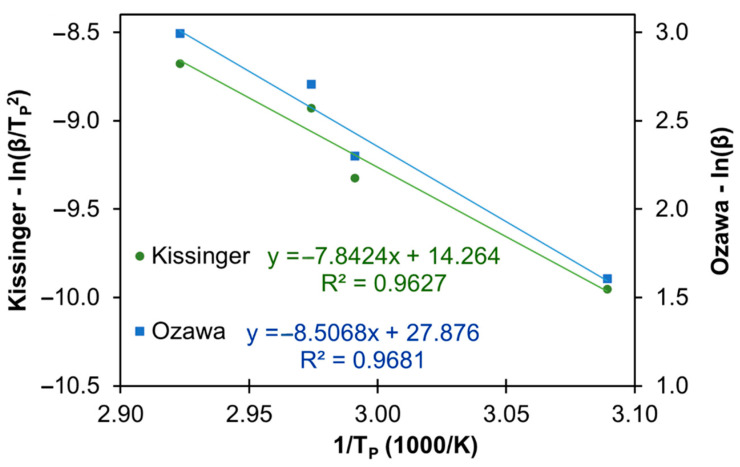
Kissinger and Ozawa plots for the determination of the *E*_a_ of the curing process of LS-based PU containing 150 μL PEG_200_ (formulation 8, Table 1).

Next, the *E*_a_ of the curing process of LS-based PU containing PEG_200_ (150 μL) was determined using the Kissinger and the Ozawa methods, and the corresponding plots are depicted in Figure 10. From the slopes of each linear plot, *E*_a_ was 65.2 and 70.7 kJ·mol^−1^, for the Kissinger and Ozawa methods, respectively. The value of *E*_a_ for the Ozawa method is higher than the one for the Kissinger method due to approximations performed in the Ozawa method, which is in agreement with the literature data [13,71]. 

**Figure 11:**
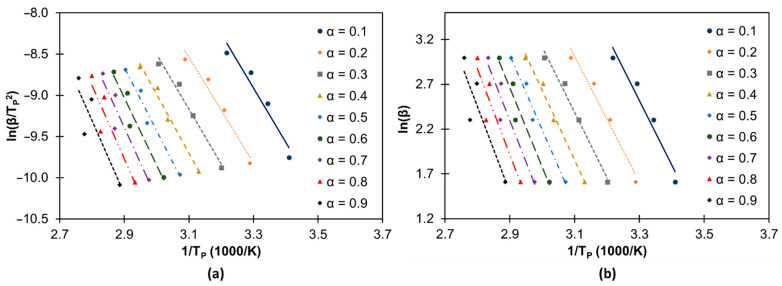
Determination of *Ea* using the Kissinger (**a**) and the Ozawa (**b**) methods for the curing process of LS-based PU containing PEG_200_ (150 μL).

**Figure 12:**
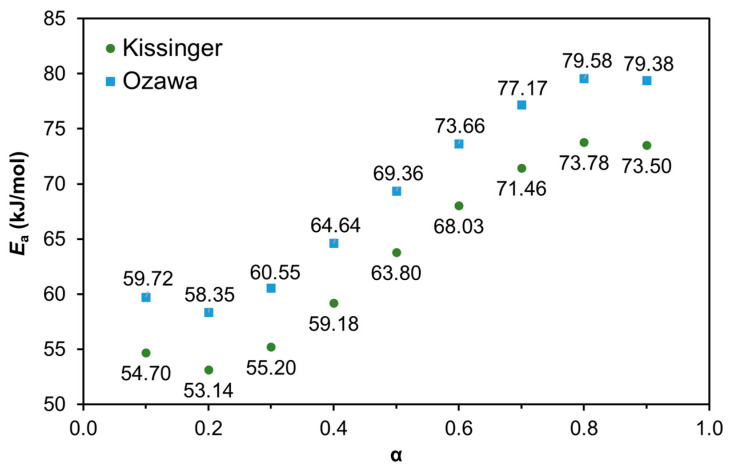
Plots representing the dependence of *E*_a_ with α using the Kissinger and the Ozawa methods.

Since *E*_a_ is not constant and varies with α, the Kissinger and the Ozawa methods can be used to determine the *E*_a_ throughout the entire cure reaction [13,69,70,71]. Therefore, the resulting Kissinger and Ozawa plots are presented in Figure 11 including the respective equations for the different α, which are listed in Table 3. The *E*_a_ values calculated for different extends of reaction using the Kissinger and the Ozawa methods are shown in Figure 12. The trend observed is in agreement with that registered in a previous study regarding the variation of *E*_a_ throughout the curing process of a castor oil-based PU [13]. As suggested in that study, the increase in *E*_a_ at lower α values (from α = 0.1 to 0.7) can be attributed to the crosslinking within the PU network while the slight decrease in *E*_a_ at higher α values can be attributed to the breaking of crosslinks resulting in increasing chain flexibility. This type of behavior was also observed for epoxy resins [71].

## 4. Conclusions

The results of this study demonstrate the possibility of using hardwood lignosulphonates as macropolyols in polyurethane formulations suitable for adhesive purposes. Due to some limitations in the accessibility/reactivity of hydroxyl groups in lignosulfonate (LS) in the reaction with 4′-methylene diphenyl diisocyanate (pMDI) and difficulties in effective homogenization of the reaction mixture, the addition of polyethylene glycol diol of low molecular weight (*M*_w_ 200, PEG_200_) seems to be advantageous to overcome, at least partially, these drawbacks. The important role of PEG_200_ in the consolidation of LS-based PU network via crosslinking reactions with pMDI has been demonstrated. It was also suggested that the addition of PEG_200_ in the LS-pMDI reaction mixture favored the interfacial interaction between the LS-based PU adhesive and the glued material (wood strips). The adhesion strength of LS-based PU was comparable to the commercial white glue. The curing kinetics of LS-based PU adhesive with addition of PEG_200_ showed similar trend to those observed previously with other PU formulations. The activation energy determined is within the range of 60–70 kJ·mol^−1^ depending the applied methodology. The next step would be to study the use of unrefined spent liquor for the same purpose.

## Figures and Tables

**Table 1:** Formulations of the LS-based PU adhesives prepared.

Formulation	LS (mg)	H_2_O (μL)	PEG_200_ (μL)	DBTDL (μL)	MDI (mg)	NCO/OH Molar Ratio *
1	500	400	0	50	900	3.2:1
2	500	400	50	50	900	2.6:1
3	500	400	100	50	900	2.2:1
4	500	400	150	50	900	1.9:1
5	500	400	0	50	1000	3.6:1
6	500	400	50	50	1000	2.9:1
7	500	400	100	50	1000	2.4:1
8	500	400	150	50	1000	2.1:1

* Without water.

**Table 2:** Assignments of the FTIR bands in the spectra of LS, LS-MDI and LS-MDI-PEG [45,55,56,57,58].

Band (cm^−1^)	Assignment
3600–3200	O–H stretching; N–H stretching (urethane group)
2908/2842	C–H stretching in –CH_2_–, –CH_3_ and O–CH_3_ groups
2262	C–N stretching (isocyanate group)
1766	C=O stretching (urethane group)
1644	C=O stretching (isocyanate group)
1590	N–H deformation (isocyanate group), lignin aromatic group
1504	N–H bending (urethane group), lignin aromatic group
1406	C–N stretching in amide (urethane group)
1302	C–N stretching (urethane group)
1216	C–N stretching (urethane group)
1036	C–O stretching in aliphatic OH
808	C–H deformation out-of-plane, aromatic ring

**Table 3:** Kissinger and Ozawa equations from plots in Figure 11.

α	Kissinger		Ozawa	
	Equation	R^2^	Equation	R^2^
0.1	y = −6.582x + 12.811	0.940	y = −7.186x + 26.232	0.950
0.2	y = −6.395x + 11.283	0.962	y = −7.022x + 24.780	0.969
0.3	y = −6.643x + 11.424	0.980	y = −7.287x + 24.974	0.983
0.4	y = −7.122x + 12.378	0.983	y = −7.779x + 25.968	0.986
0.5	y = −7.677x + 13.604	0.967	y = −8.346x + 27.228	0.972
0.6	y = −8.186x + 14.712	0.934	y = −8.864x + 28.365	0.943
0.7	y = −8.599x + 15.545	0.889	y = −9.286x + 29.224	0.903
0.8	y = −8.879x + 15.979	0.839	y = −9.576x + 29.686	0.858
0.9	y = −8.845x + 15.459	0.773	y = −9.552x + 29.197	0.799

## Data Availability

Data sharing not applicable.

## Supplementary Materials

The following are available online at https://www.mdpi.com/article/10.3390/ma14227072/s1, Figure S1: TGA curve of PEG200, under inert N_2_ gas flow.
