# Supplementary material for: Lignosulfonate-Based Polyurethane Adhesives
Source: Materials (Basel). 2021 Nov 21;14(22):7072. doi: 10.3390/ma14227072 (PMC8622556; doi:10.3390/ma14227072)
Supplement: Supplementary file 1 [file materials-14-07072-s001.zip › materials-1463368-supplementary.pdf]

materials

MDPI

Supplementary Materials

Lignosulfonate-Based Polyurethane Adhesives

Sandra Magina [1], Nuno Gama [1], Luísa Carvalho [2], Ana Barros-Timmons [1] and Dmitry V. Evtuguin [1,*]

[1] CICECO and Department of Chemistry, University of Aveiro, 3810-193 Aveiro, Portugal;
smagina@ua.pt (S.M.); nuno.gama@ua.pt (N.G.); anabarros@ua.pt (A.B.-T.)

[2] LEPABE—Laboratory for Process, Environmental and Energy Engineering, Faculdade de Engenharia,
University of Porto, 4200-465 Porto and DEMad—Department of Wood Engineering,
Instituto Politécnico de Viseu, 3504-510 Viseu, Portugal; lhcarvalho@estv.ipv.pt

* Correspondence: dmitrye@ua.pt

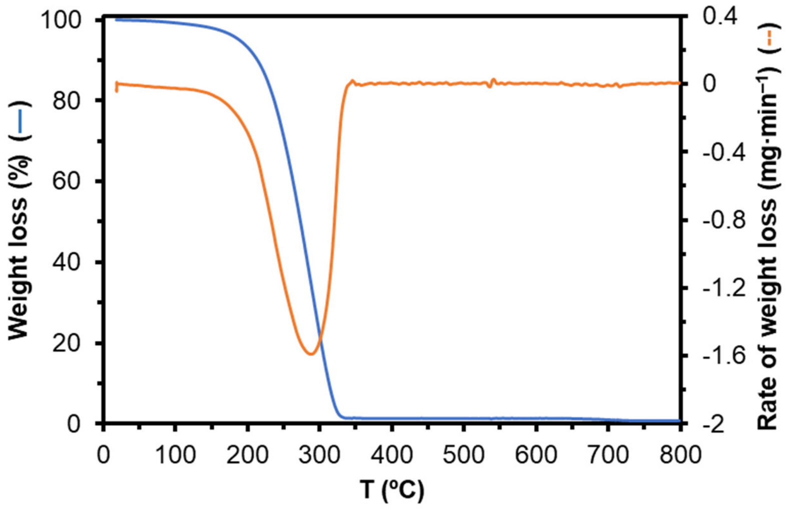

Figure S1. TGA curve of PEG_{200}, under inert N_2 gas flow.
